# Neurofilament Light Chain Is Associated With Acute Mountain Sickness

**DOI:** 10.1002/brb3.70165

**Published:** 2024-11-17

**Authors:** Klaus Berek, Anna Lindner, Franziska Di Pauli, Gabriel Bsteh, Benedikt Treml, Markus Ponleitner, Clemens Engler, Axel Kleinsasser, Thomas Berger, Maria Wille, Martin Burtscher, Florian Deisenhammer, Harald Hegen

**Affiliations:** ^1^ Department of Neurology Medical University of Innsbruck Innsbruck Austria; ^2^ Department of Neurology Medical University of Vienna Vienna Austria; ^3^ Comprehensive Center for Clinical Neurosciences and Mental Health Medical University of Vienna Vienna Austria; ^4^ Department of Anaesthesiology and Critical Care Medicine Medical University of Innsbruck Innsbruck Austria; ^5^ Department of Surgery University Hospital for Cardiac Surgery Medical University of Innsbruck Innsbruck Austria; ^6^ Department of Sport Science University of Innsbruck Innsbruck Austria

**Keywords:** acute mountain sickness, biomarker, headache, high altitude, neurofilament light chain

## Abstract

**Background:**

Neurological symptoms are common in acute mountain sickness (AMS); however, the extent of neuroaxonal damage remains unclear. Neurofilament light chain (NfL) is an established blood biomarker for neuroaxonal damage.

**Objective:**

To investigate whether plasma (p) NfL levels increase after simulated altitude exposure, correlate with the occurrence of AMS, and might be mitigated by preacclimatization.

**Methods:**

Healthy subjects were exposed to simulated high altitude (4500 m) by the use of a normobaric hypoxic chamber at the University of Innsbruck two times, that is, within Cycle 1 (C1) over 12 h, and within Cycle 2 (C2) for another 12 h but with a random assignment to prior acclimatization or sham acclimatization. Before each cycle (measurement [M] 1 and 3) and after each cycle (M2 and M4), clinical data (arterial oxygen saturation [SaO_2_], heart rate, and Lake Louise AMS score [LLS]) and plasma samples were collected. pNfL was measured using single‐molecule array (Simoa) technique.

**Results:**

pNfL levels did not significantly change within each study cycle, but increased over the total study period (M1: 4.57 [3.34–6.39], M2: 4.58 [3.74–6.0], M3: 5.64, and M4: 6.53 [4.65–7.92] pg/mL, *p* < 0.001). Subjects suffering from AMS during the study procedures showed higher pNfL levels at M4 (6.80 [6.19–8.13] vs. 5.75 [4.17–7.35], *p* = 0.048), a higher total pNfL increase (2.88 [1.21–3.48] vs. 0.91 [0.53–1.48], *p* = 0.022) compared to subjects without AMS. An effect of preacclimatization on pNfL levels could not be observed.

**Conclusions:**

pNfL increases alongside exposure to simulated altitude and is associated with AMS.

## Graphical abstract

Healthy subjects were exposed to simulated high altitude (4500 m) two times, i.e., within Cycle 1 and Cycle 2, the latter with a random assignment to prior acclimatization or sham acclimatization. Before and after each cycle, pNfL was measured. pNfL levels increased over the total study period. Subjects suffering from AMS during the study procedures showed higher pNfL levels at M4 and a higher total pNfL increase.

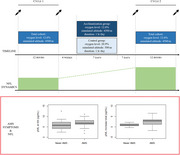

## 1 Introduction

High‐altitude regions are physically and psychologically demanding environments for visitors. The rapid ascent to high altitudes is associated with the risk of high‐altitude illnesses (Wilson, Newman, and Imray 2009; Luks, Swenson, and Bärtsch 2017), a heterogeneous compilation of disease entities of which acute mountain sickness (AMS) represents the most common one (Netzer et al. 2013). The leading symptom of AMS is headache, which may be accompanied by low appetite, nausea, fatigue, weakness, and dizziness (Roach et al. 2018). Classical risk factors for AMS include the absolute altitude reached, ascent rate, degree of acclimatization, and individual susceptibility (Mairer et al. 2009; Jarius et al. 2012).

Mostly, AMS is self‐limiting and resolves without treatment. However, it may evolve to a more severe form such as high altitude cerebral edema (HACE) (Hackett and Roach 2001), which usually presents with headache, decreased consciousness, and truncal ataxia and may rapidly progress to coma or death without appropriate and prompt therapy (Luks, Swenson, and Bärtsch 2017; Hackett et al. 1998). In patients with HACE, magnetic resonance imaging (MRI) studies showed edema in the corpus callosum region and evidence of blood–brain barrier leakage (Hackett et al. 1998; Schommer et al. 2013). However, MRI detected slight brain swelling also in mildly affected or even asymptomatic subjects (Kallenberg et al. 2007). Therefore, it may be hypothesized that neuronal damage may occur in asymptomatic subjects when acutely exposed to simulated high altitudes. Nevertheless, a robust marker to measure subclinical affection of the central nervous system in AMS is still scarce. Obviously, a peripheral body fluid marker would be more practicable than possible MRI markers.

Neurofilament light chain (NfL) is part of the cytoskeleton of neuronal structures and an established biomarker of neuroaxonal damage (Khalil et al. 2018). It has proven its diagnostic and prognostic potential in a broad spectrum of disorders affecting the central nervous system, for example, in multiple sclerosis (Disanto et al. 2017; Novakova et al. 2017), traumatic brain injury (Shahim et al. 2017; Shahim et al. 2016; Al Nimer, Thelin, and Nystrom 2015), but also in brain hypoxia (Hoiland et al. 2021).

In the present study, we investigated whether plasma NfL (pNfL) levels increase during and after exposure to normobaric hypoxia, whether pNfL correlates with the occurrence of AMS, and whether an increase in pNfL levels might be mitigated by preacclimatization.

## 2 Methods

A detailed description of the study design has been previously published; only remaining samples from this study were analyzed (Treml et al. 2020). Briefly, physically fit, healthy subjects were eligible for inclusion. Exclusion criteria subsumed history of cardiac, pulmonary, psychiatric, or neurological illnesses, as well as preceding exposure to altitude, that is, visiting heights ≥ 2500 m for ≥ 24 h within the last 4 weeks prior to the study, or permanently living ≥ 1000 m.

All healthy study participants were exposed to simulated altitude in a normobaric hypoxic chamber located at the Department of Sports Science, Leopold‐Franzens University, Innsbruck, Austria, two times (Cycle 1 and Cycle 2), each for 12 h, with an oxygen level of 12.6% being the equivalent of a height of approximately 4500 m. Before each cycle (i.e., measurement [M]1 and M3) and after each cycle (M2 and M4), anthropometric and clinical characteristics (body weight and height, heart rate, systemic blood pressure, arterial oxygen saturation, and severity of AMS) were assessed, and a blood sample (EDTA‐plasma) was drawn by peripheral venous puncture. Clinical parameters were measured in a sitting position 3 h after initiation of hypoxia. AMS severity was measured using the 2018 revised Lake Louise Acute Mountain Sickness Score (LLS) ranging from 0 (no symptoms) to 12 (severe AMS symptoms) (Roach et al. 2018). LLS was measured repeatedly during study procedures (Cycles 1 and 2), and the highest values of LLS were recorded at each study cycle. The presence of AMS was defined as an LLS ≥ 4 (Roach et al. 2018; Treml et al. 2020). As indicated below, subjects were stratified into a “Never AMS group” and an “AMS group” according to the occurrence of AMS at least once during both study cycles.

**FIGURE 1:**
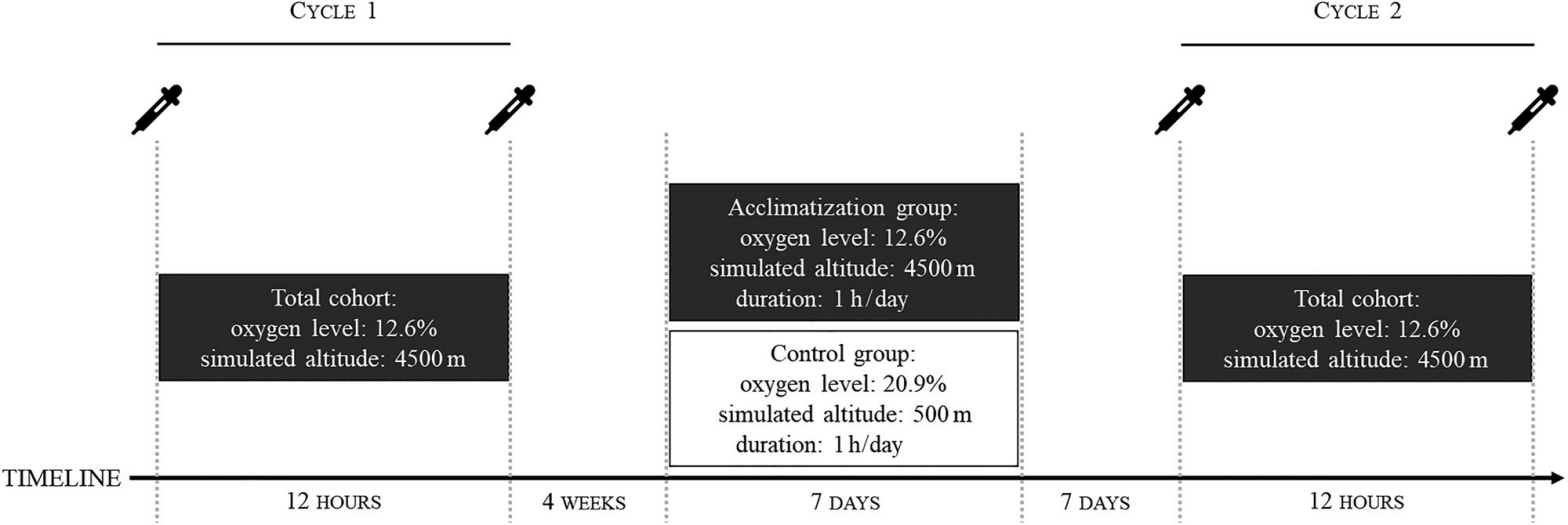
Study‐related procedures. Pipettes indicate sampling time‐points.

After Cycle 1 and a deconditioning phase of 4 weeks, subjects were randomly assigned to an acclimatization or sham acclimatization group, that is, the control group. Both groups were blinded for their group assignment. The acclimatization group was exposed to a preacclimatization protocol consisting of exposure to an oxygen level of 12.6% for 1 h at 7 consecutive days. The control group was exposed to an oxygen level of 20.9%, indicating sham acclimatization for 1 h at 7 consecutive days. Before Cycle 2, another deconditioning phase of 7 days was scheduled. All procedures of Cycle 1 were repeated at Cycle 2. A graphical overview of study procedures is given in Figure 1.

### 2.1 Sample Procession and NfL Measurement

All samples were stored at −20°C from the time‐point of collection until NfL measurement. pNfL levels were measured at the Department of Neurology, Medical University of Vienna. Study personnel conducting the measurement was blinded for demographic and clinical data as well as group assignment. Measurements were performed using the single‐molecule enzyme‐linked immunosorbent assay (Simoa) technique on a Simoa SR‐X Analyzer (Quanterix, Lexington, MA, USA) (Rissin et al. 2010). For all measurements, the manufacturer's instructions and protocol were adhered to.

As NfL concentrations increase with age and decrease with BMI under physiological conditions, we calculated age‐ and BMI‐adjusted *Z* scores. This allows to quantify the deviation of each patient's individual pNfL value in comparison to control persons of the same age and BMI, based on a recently published reference database (Benkert et al. 2022).

### 2.2 Statistical Analysis

Statistical analysis was performed using R software (Benkert et al. 2022). Data were checked for normal distribution using the Shapiro–Wilk test. Data are shown as mean ± standard deviation (SD), or as median and interquartile range (IQR), as appropriate. Group comparisons were performed by the Mann–Whitney *U* test or *χ* ^2^ test as appropriate. Repeated measurements were analyzed by Friedman, Wilcoxon test, and ANOVA, respectively. Pairwise complete observation units were used for repeated measurement analyses. According to the occurrence of AMS, subjects were stratified into a “Never AMS group” and an “AMS group,” indicating an LLS ≥ 4 at least once during the two study cycles. Two‐sided *p*‐values < 0.05 were considered statistically significant.

## 3 Results

A total of 63 healthy subjects at a median age of 24 (IQR 22–28) years were included in the study. Twenty‐seven (43%) participants were female, and the median BMI was 22 (21–24). A detailed description of demographics, clinical, and laboratory characteristics is given in Table 1.

**TABLE 1:** Demographic, clinical, and laboratory characteristics of participants.

Number of participants (*n* = 63)				
Sex (female)	27 (43)			
Age (years)	24 ± 5			
BMI (kg/m^2^)	22 (21–24)			

Clinical and laboratory data of participants				
	M1	M2	M3	M4
Number of samples	51	48	44	43
pNfL (pg/mL)	4.57 (3.34–6.39)	4.58 (3.74–6.0)	5.64 (4.46–8.04)	6.53 (4.65–7.92)
pNfL increase (pg/mL)^a^	0.095 (−0.875 to 0.519)		0.164 (−1.117 to 1.614)	
Total pNfL increase^b^	1.70 (0.78–3.15)			
NfL *Z* score	−0.28 (−1.28 to 0.67)	−0.28 (−0.88 to 0.50)	0.45 (−0.28 to 1.29)	0.74 (−0.18 to 1.17)
NfL *Z* score increase^a^	0.16 (−0.38 to 0.50)		0.08 (−0.43 to 0.50)	
Total NfL *Z* score increase^b^	1.00 (0.53–1.65)			
LLS	4 (2–5)		2 (1–4)	
Heart rate (bpm)	80 (73–85)	84 (76–92)^b^	80 (73–86)	84 (76–92)^b^
Heart rate increase^c^	6 (−2 to 16)		6 (−2 to 15)	
SaO_2_ (%)	98 (97–98)	83 (79–87)^b^	97 (97–98)	83 (80–87)^b^
SaO_2_ decrease^c^	14 (12–18)		14 (11–16)	

*Note*: Data are depicted as median (IQR), mean ± standard deviation, and *n* (%), as appropriate. Demographic data were assessed at baseline. LLS was assessed according to Roach et al. (2018).

Abbreviations: bpm = beats per minute; LLS = Lake Louise Acute Mountain Sickness Score; M1–4 = measurements 1–4; pNfL = plasma neurofilament light; SaO_2_ = arterial oxygen saturation.

^a^ An increase/decrease of variables between M1/M2 and M3/M4, respectively.

^b^ An increase/decrease of variables between M1 and M4.

^c^ M2 and M4 of clinical parameters, that is, heart rate and SaO_2_ were performed after 3 h.

### 3.1 Exposure to Simulated Altitude and Physiological Impact

In both study cycles, heart rates of the participants significantly increased 3 h after exposure to simulated high altitude (M1: 80 [73–85] vs. M2: 84 [76–91], *p* = 0.015 and M3: 80 [73–86] vs. M4: 84 [76–92], *p* = 0.015), while median SaO_2_ levels decreased, respectively (M1: 98 [97–98] vs. M2: 83 [79–87], *p *< 0.001 and M3: 97 [97–98] vs. M4: 83 [80–87], *p* < 0.001). The median increase of the heart rate and decrease of SaO_2_ after 3 h did not differ significantly between the two cycles (6 [−2 to 16] vs. 6 [−2 to 15], *p* = 0.917, and 14 [12–18] vs. 14 [11–16], *p* = 0.280). LLS was significantly lower in Cycle 2 (2 [1–4]) than in Cycle 1 (4 [2–5], *p* < 0.001).

pNfL levels did not significantly change within the cycles: Cycle 1, M1: 4.57 [3.34–6.39] vs. M2: 4.58 [3.74–6.0], *p* = 0.783, and Cycle 2, M3: 5.64 [4.46–8.04] vs. M4: 6.53 [4.65–7.92], *p* = 0.604. However, pNfL concentrations significantly increased over the whole study period (*p* < 0.001), and the median total pNfL increase from M1 to M4 was 1.70 [0.78–3.15]. Similarly, NfL *Z* scores did not significantly increase within Cycle 1 (M1: −0.28 [−1.28 to 0.67] vs. M2: −0.28 [−0.88 to 0.50], *p* = 0.950) or within Cycle 2 (M3: 0.45 [−0.28 to 1.29] vs. M4: 0.74 [−0.18 to 1.17], *p* = 0.527), but NfL *Z* scores increased from M1 to M4. The median total NfL *Z* score increase was 1.00 (0.53–1.65, *p* = 0.015).

**FIGURE 2:**
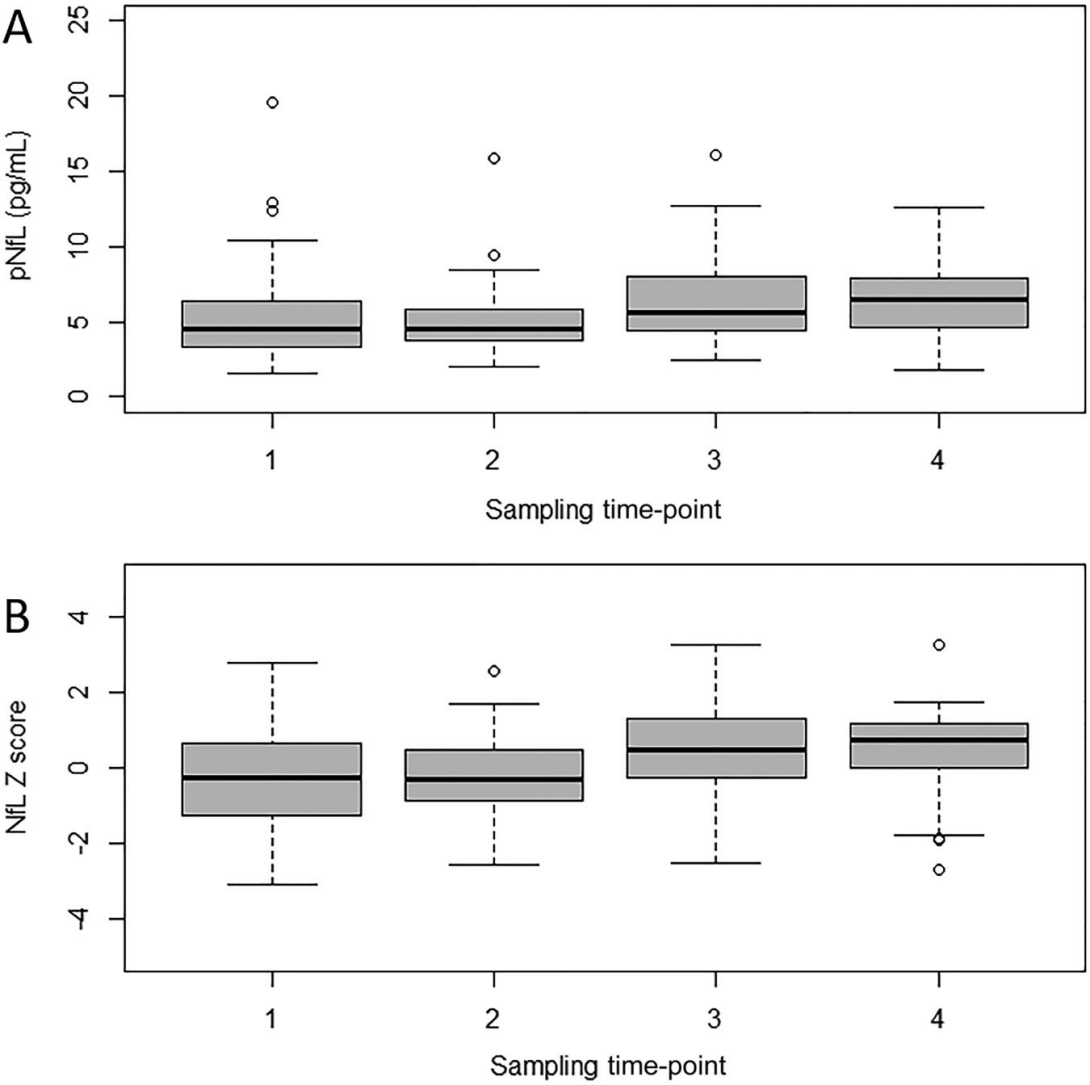
Longitudinal plasma NfL levels and NfL *Z* scores in subjects before and after altitude exposure. The *X*‐axis depicts the different sampling time‐points; sampling time‐points 1 and 2 before and after study Cycle 1, sampling time‐points 3 and 4 before and after study Cycle 2. NfL = neurofilament light.

A graphical overview of pNfL levels at different time‐points of measurement is given in Figure 2.

### 3.2 Clinical Factors and NfL Are Associated With the Occurrence of AMS

Subjects of the “AMS” group were significantly younger (25 ± 5 vs. 27 ± 5 years, *p* = 0.037) and showed a larger decrease of SaO_2_ (15 [11–17] vs. 13 [9–15], *p *< 0.001) and a more pronounced heart rate increase (7 [0–17] vs. 2 [−10 to 13], *p* = 0.004) compared to those of the “Never AMS” group.

No differences between the “Never AMS group” and the “AMS group” could be identified at baseline and at second measurement of Cycle 1 for absolute pNfL levels and NfL *Z* scores (M1: pNfL: 4.81 [3.99–7.35] vs. 4.31 [3.04–5.74], *p* = 0.233, NfL *Z* score: −0.71 [−1.48 to 0.05] vs. −0.01 [−0.99 to 1.01], *p* = 0.098; M2: pNfL: 4.66 [3.85–6.15] vs. 4.50 [3.34–5.85], *p* = 0.319, NfL *Z* score: −0.67 [−1.64 to 0.25] vs. 0.13 [−0.81 to 0.52], *p* = 0.058).

At Cycle 2, pNfL levels (M3: 6.69 [5.57–8.78] vs. 4.77 [3.89–8.01], *p* = 0.086; and M4: 6.80 [6.19–8.13] vs. 5.75 [4.17–7.35], *p* = 0.048) and NfL *Z* scores (M3: −0.15 [−1.17 to 0.50] vs. 0.74 [0.00–1.34], *p* = 0.022; and M4: 0.25 [−0.95 to 1.08] vs. 1.02 [0.62–0.50], *p* = 0.011) were higher in subjects suffering from AMS compared to those who did not.

**FIGURE 3:**
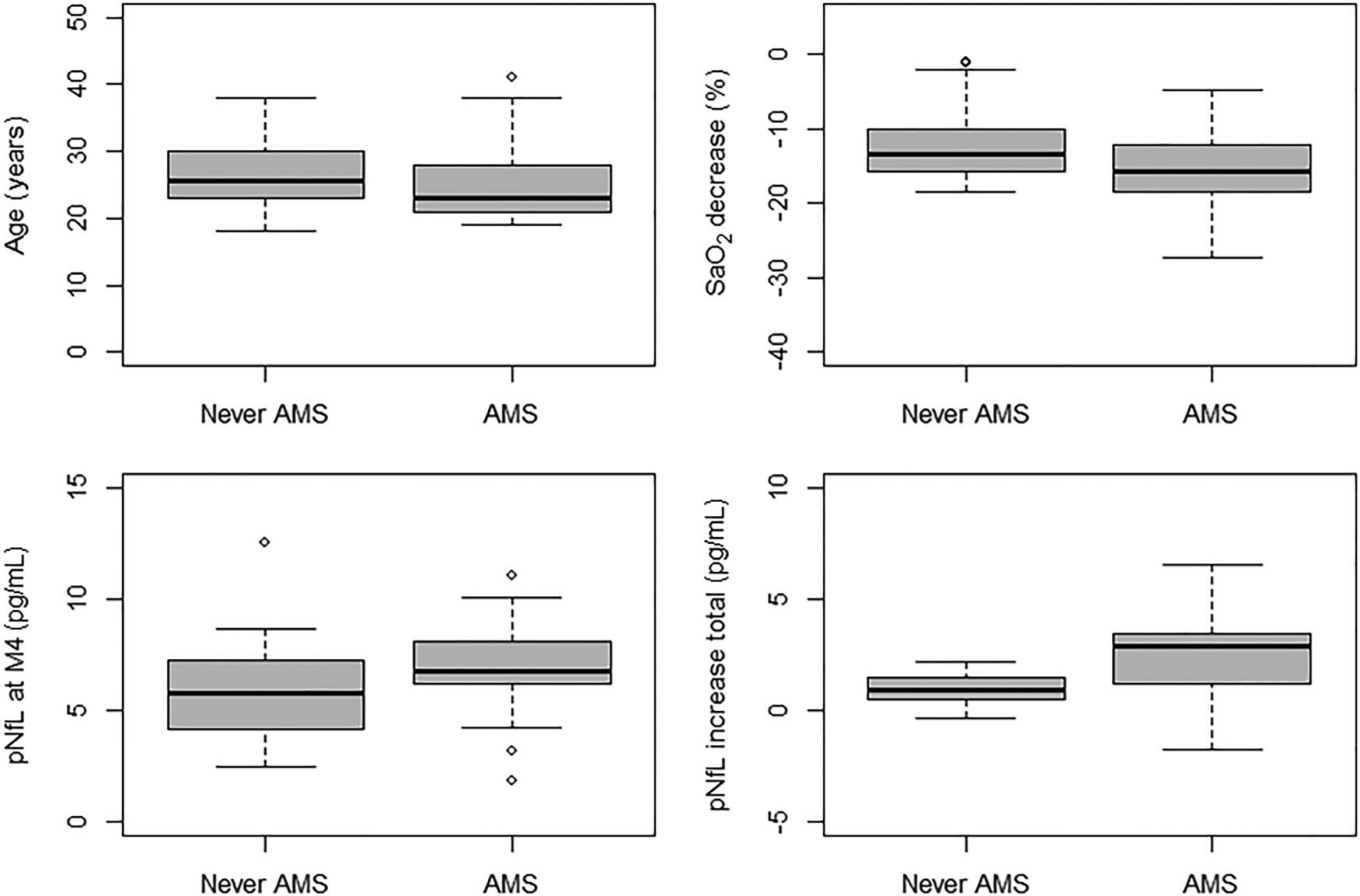
Factors associated with the occurrence of AMS. LLS was assessed according to (Roach et al. 2018). “SaO_2_ decrease” is the decrease of SaO_2_ 3 h after exposure to simulated high altitude. “pNfL increase total” indicates the increase of pNfL from M1 to M4. AMS = acute mountain sickness; LLS = Lake Louise Acute Mountain Sickness Score; M1/M4 = measurement 1/4; pNfL = plasma neurofilament light; SaO_2_ = arterial oxygen saturation.

The total increase of pNfL (2.88 [1.21–3.48] vs. 0.91 [0.5321.48], *p* = 0.022) and of NfL *Z* scores (0.78 [0.32–1.00] vs. 1.56 [0.53–2.45], *p* = 0.072) from M1 to M4 was higher in subjects of the “AMS group” than in those of the “Never AMS group.” The repeated measures ANOVA also revealed a significantly higher pNfL increase in the “AMS group” than in the “Never AMS group” (*p* < 0.001). Differences in demographic, clinical, and laboratory characteristics according to the “AMS” and the “Never AMS group” are displayed in Table 2 and Figure 3.

**TABLE 2:** Differences of probands' characteristics according to the occurrence of AMS.

Clinical and laboratory characteristics			
	Never AMS group^a^	AMS group	*p* value
Age (years)	27 ± 5	25 ± 5	**0.037**
BMI (kg/m^2^)	22 (21–24)	22 (21–24)	0.412
Heart rate increase^b^	2 (−10 to 13)	7 (0–17)	**0.004**
SaO_2_ decrease^b^	13 (9–15)	15 (11–17)	**< 0.001**
Sample number at M1	19	32	n.a.
pNfL at M1 (pg/mL)	4.31 (3.04–5.74)	4.81 (3.99–7.35)	0.233
NfL *Z* score M1	−0.71 (−1.48 to 0.05)	−0.01 (−0.99 to 1.01)	0.098
Sample number at M2	18	30	n.a.
pNfL at M2 (pg/mL)	4.50 (3.34–5.85)	4.66 (3.85–6.15)	0.319
NfL *Z* score M2	−0.67 (−1.64 to 0.25)	0.13 (−0.81 to 0.52)	0.058
pNfL increase C1^b^	0.27 (−0.18 to 0.50)	−0.17 (−1.40 to 0.52)	0.163
NfL *Z* score increase C1^b^	0.26 (−0.21 to 0.56)	−0.12 (−0.74 to 0.46)	0.125
Sample number at M3	19	25	n.a.
pNfL at M3 (pg/mL)	4.77 (3.89–8.01)	6.69 (5.57–8.78)	0.086
NfL *Z* score M3	−0.15 (−1.17 to 0.50)	0.74 (0.00–1.34)	**0.022**
Sample number at M4	19	24	n.a.
pNfL at M4 (pg/mL)	5.75 (4.17–7.35)	6.80 (6.20–8.13)	**0.048**
NfL *Z* score M4	0.25 (−0.95 to 1.08)	1.02 (0.62–0.50)	**0.011**
pNfL increase C2^b^	0.16 (−0.81 to 1.19)	0.19 (−1.23 to 1.71)	0.990
NfL *Z* score increase C2^b^	0.08 (−0.45 to 0.22)	0.07 (−0.41 to 0.50)	0.807
pNfL increase total^c^	0.91 (0.53–1.48)	2.88 (1.21–3.48)	**0.022**
NfL *Z* score increase total	0.78 (0.32–1.00)	1.56 (0.53–2.45)	0.072

*Note*: Data are depicted as median (IQR), mean ± standard deviation, as appropriate. Group comparisons were performed by the Mann–Whitney *U* test. *p*‐values < 0.05 were considered statistically significant and are marked bold.

Abbreviations: AMS = acute mountain sickness; bpm = beats per minute; C1 = Cycle 1; C2 = Cycle 2; LLS = Lake Louise Acute Mountain Sickness Score; M1–4 = measurements 1–4; n.a. = not applicable; pNfL = plasma neurofilament light; SaO_2_ = arterial oxygen saturation.

^a^ AMS was defined as LLS ≥ 4. LLS was assessed according to Roach et al. (2018).

^b^ An increase/decrease of variables within the respective cycle.

^c^ The total increase of pNfL from M1 to M4.

### 3.3 The Effect of Preacclimatization on pNfL Levels

Absolute pNfL levels at M3 (5.20 [4.34–7.84] vs. 6.89 [5.03–8.77], *p* = 0.203) and M4 (6.53 [4.18–7.33] vs. 6.89 [5.21–8.83], *p* = 0.242) did not differ significantly between the control group and the acclimatization group. The pNfL increase during cycle 2 (0.16 [−1.34 to 1.87] vs. 0.18 [−0.90 to 1.40], *p* = 0.884) did not show significant differences between the control group and the acclimatization group. pNfL levels and increases according to the control and acclimatization groups are depicted in Table S1.

## 4 Discussion

In the present study, we performed longitudinal measurements of pNfL levels after exposure to simulated high altitude in healthy subjects and revealed two main findings: (i) NfL levels increase with exposure to simulated altitude, (ii) the occurrence of AMS is associated with higher NfL levels after repeated exposure to simulated altitude (M4).

The visit to high altitudes for various reasons is a globally increasing trend being connected to potentially severe health risks (Burtscher, Hefti, and Hefti 2021). Depending on risk factors such as absolute height, speed of ascent, individual susceptibility, and lack of acclimatization, persons exposed to high altitude can develop AMS or even HACE (Wilson, Newman, and Imray 2009; Luks, Swenson, and Bärtsch 2017; Roach et al. 2018; Mairer et al. 2009; Schneider et al. 2002). Thus, permanent damage to the CNS may be a potential risk of exposure to high altitudes. Such CNS damage is likely to be reflected by elevated NfL levels, as NfL represents an established robust biomarker with high sensitivity for any occurring neuroaxonal damage (Khalil et al. 2018). The evident potential of NfL to monitor neurological damage after high‐altitude exposure has so far only been investigated in one prior study (Sareban et al. 2021) reporting significantly increased NfL levels in healthy volunteers, 44 h after reaching 4559 m; however, they did not find any relation to AMS or extent of hypoxemia. In line with this, our data suggest an increase of pNfL with increasing time exposed to high altitude. Furthermore, we could demonstrate a relation between pNfL levels and the occurrence of AMS. However, also in our cohort, a significant pNfL increase and its relation to the occurrence of AMS were only observed if the total study time was assessed (M1–M4), not within each cycle. Therefore, in the previous study, pNFL might have been measured too early, or too few cycles of high‐altitude exposure may have been applied to capture this association. This may also be seen in line with literature from other neurological diseases, in which hypoxia plays a crucial role, that is, in acute ischemic stroke, a significant NfL increase has been reported within days with its maximum approximately 3 months after onset (Pedersen et al. 2019; Tiedt et al. 2018; Gattringer et al. 2017). From a mechanistic point of view, it may therefore be hypothesized that after neuroaxonal damage caused by hypoxia, it takes days to months to set NfL free. This may also have influenced our negative finding concerning the effect of the preacclimatization protocol on pNfL levels, as we had only a very short follow‐up time after the preacclimatization protocol. The true NfL peak may have occurred substantially later than M4.

Further factors associated with AMS in our cohort were a larger decrease of SaO_2_ after 3 h in simulated high altitude and younger age. The former is in line with earlier reports, suggesting the level of SaO_2_ decrease after acute exposure to high altitude to be a predictor of the occurrence of AMS (Burtscher et al. 2019). The latter may enrich the academic discourse on the hypothesis that younger age may be a risk factor for AMS (Honigman et al. 1993). This discussion is connected to the historical, however, recently challenged so‐called “tight‐fit” hypothesis (Ross 1985). Our data in fact may be of special interest in this context and other theories on pathophysiological reasons for neurological symptoms at high altitudes. For years, the prevailing concept was that the interindividual difference in AMS susceptibility is the result of different “tightness” of the brain in the cranial vault, that is, a higher brain‐volume‐to‐intracranial‐volume ratio in susceptible persons (Ross 1985). This hypothesis has recently been challenged by findings of venous hypertension and molecular mechanisms contributing to the occurrence and severity of AMS and HACE (Wilson, Newman, and Imray 2009). The consequently proposed “modified tight‐fit” hypothesis takes also into account these factors and suggests that neurological symptoms may be caused or aggravated by the rise of intracranial pressure by increasing the volume of cranial contents or failure to buffer. The former may subsume anatomical susceptibility, increased intracranial blood volume, or edema (cytotoxic and/or vasogenic), and the latter failures in the two main buffer systems: cerebrospinal fluid (CSF) and venous outflow (Wilson, Newman, and Imray 2009). This theory is underpinned by measurements of optic nerve sheath diameter, indicating an indirect measurement of the intracranial pressure, which are associated with the occurrence and intensity of AMS (Fagenholz et al. 2009; Kanaan et al. 2015).

Against this background, we provide data of higher pNfL levels and larger pNfL increase in subjects suffering from AMS. Therefore, we further substantiate the “modified tight‐fit” hypothesis: The more increase of intracranial pressure due to high altitude, the more (neurological) AMS symptoms and the more pNfL increase. Eventually, hypoxia is associated with both NfL increase (Hoiland et al. 2021) and AMS severity (Burtscher, Flatz, and Faulhaber 2004). Measuring absolute NfL levels or—even better—NfL increase is therefore very likely to be closely related to measuring hypoxia. By demonstrating a link between NfL and AMS, we kind of “close the circle” and provide a biomarker to quantify the neuronal damage in relation to AMS caused by hypoxia.

There are some limitations of our study. First, all measurements were performed before, during, or after exposure to simulated high altitude by the use of a normobaric hypoxic chamber. Indeed, our results may rather display an association of pNfL and hypoxia than between pNfL and high‐altitude exposure. Nonetheless, the essential physiological characteristic of high altitude is the reduction of oxygen partial pressure, leading to hypoxia. Key biological adaptations to high altitude are based on the degree of hypoxia. Furthermore, it has been demonstrated that normobaric hypoxia is a robust model for real (hypobaric) high‐altitude exposure (Burtscher, Flatz, and Faulhaber 2004), although it still remains a model. In actual exposure to high altitude, physical conditions such as weather, cold, solar radiation, physical exhaustion, and dehydration may have a further impact and therefore may limit the generalizability of our findings. In terms of study standardization, however, this may also be seen as a strength, as potential confounding effects, as mentioned above, were ruled out in our cohort. The absolute increases of pNfL in our study were small and, therefore, the clinical relevance may be questioned. Some of the low effect size may be explained by our study using EDTA plasma samples, since it is known that absolute levels of NfL are lower if measured in plasma than in serum (Altmann et al. 2021). More importantly, this study was not primarily designed to reveal clinical implications of pNfL increases, that is, possible neurological long‐term impacts of high altitude. It was designed as a proof of concept, whether pNfL increases in simulated high altitude and might contribute to the pathophysiological concepts of AMS.

In conclusion, we provide evidence that pNfL levels increase during exposure to simulated altitude and are associated with the occurrence of AMS.

## Author Contributions

**Klaus Berek**: conceptualization, funding acquisition, writing–original draft, methodology, visualization, formal analysis, project administration, resources, data curation, software. **Anna Lindner**: conceptualization, data curation, project administration, resources, visualization, writing–review and editing. **Franziska Di Pauli**: conceptualization, methodology, writing–review and editing. **Gabriel Bsteh**: formal analysis, investigation, methodology, resources, validation, writing–review and editing. **Benedikt Treml**: investigation, writing–review and editing, project administration, resources, data curation. **Markus Ponleitner**: investigation, writing–review and editing, resources. **Clemens Engler**: investigation, writing–review and editing, resources. **Axel Kleinsasser**: data curation, investigation, project administration, resources, writing–review and editing. **Thomas Berger**: conceptualization, methodology, writing–review and editing. **Maria Wille**: investigation, resources, writing–review and editing. **Martin Burtscher**: conceptualization, data curation, investigation, methodology, resources, supervision, validation, writing–review and editing. **Florian Deisenhammer**: conceptualization, data curation, methodology, resources, supervision, validation, writing–review and editing. **Harald Hegen**: conceptualization, formal analysis, methodology, resources, supervision, validation, visualization, writing–original draft, writing–review and editing.

## Ethics Statement

The study was approved by the ethics committee of the Medical University of Innsbruck (approval number 1130/2022). Written informed consent was obtained from all participants.

## Conflicts of Interest

Klaus Berek has participated in meetings sponsored by and received travel funding or speaker honoraria from Roche, Teva, Merck, Biogen, Sanofi and Novartis. He is associate editor of Frontiers in Immunology/Neurology, Section Multiple Sclerosis and Neuroimmunology. Franziska Di Pauli has participated in meetings sponsored by, received honoraria (lectures, advisory boards, and consultations) or travel funding from Bayer, Biogen, Merck, Novartis, Sanofi‐Genzyme, Teva, Celgene and Roche. Gabriel Bsteh has participated in meetings sponsored by, received speaker honoraria or travel funding from Biogen, Celgene/BMS, Janssen, Lilly, Merck, Novartis, Roche, Sanofi‐Genzyme and Teva, and received honoraria for consulting Biogen, Celgene/BMS, Janssen, Novartis, Roche, Sanofi‐Genzyme and Teva. He has received unrestricted research grants from Celgene/BMS and Novartis. Benedikt Treml received speaker honoraria or travel funding from AOP Orphan, Pfizer and Fresenius. Markus Ponleitner has participated in meetings sponsored by, received speaker or consulting honoraria from Amicus and travel funding from Amicus, Merck, Novartis and Sanofi‐Genzyme. Thomas Berger has participated in meetings sponsored by and received honoraria (lectures, advisory boards, consultations) from pharmaceutical companies marketing treatments for MS: Allergan, Bayer, Biogen, Bionorica, Biologix, BMS/Celgene, Eisai, Janssen‐Cilag, Jazz/GW, Horizon, MedDay, Merck, Novartis, Octapharma, Roche, Sandoz, Sanofi‐Genzyme, UCB, Teva. His institution has received financial support in the past 12 months by unrestricted research grants (Biogen, Bayer, BMS/Celgene, Merck, Novartis, Sanofi Aventis, Teva and for participation in clinical trials in multiple sclerosis sponsored by Alexion, Bayer, Biogen, Merck, Novartis, Octapharma, Roche, Sanofi‐Genzyme, Teva. Florian Deisenhammer has participated in meetings sponsored by or received honoraria for acting as an advisor/speaker for Alexion, Almirall, Biogen, Celgene, Merck, Novartis, Roche and Sanofi‐Genzyme. His institution received scientific grants from Biogen and Sanofi‐Genzyme. Harald Hegen has participated in meetings sponsored by, received speaker honoraria or travel funding from Bayer, Biogen, Bristol Myers Squibb, Horizon, Janssen, Merck, Novartis, Sanofi‐Genzyme, Siemens, Teva, and received honoraria for acting as consultant for Biogen, Bristol Myers Squibb, Novartis, Roche, Sanofi‐Genzyme and Teva. He is associate editor of Frontiers in Neurology. The other authors declare no conflicts of interest.

### Peer Review

The peer review history for this article is available at https://publons.com/publon/10.1002/brb3.70165.

## Supporting information



Supporting Information

## Data Availability

Anonymized data will be shared upon reasonable request from any qualified investigator and upon approval by the data‐clearing committee of the Medical University of Innsbruck.

## References

[brb370165-bib-0001] Al Nimer, F. , E. Thelin , and H. Nystrom , et al. 2015. “Comparative Assessment of the Prognostic Value of Biomarkers in Traumatic Brain Injury Reveals an Independent Role for Serum Levels of Neurofilament Light.” PLoS ONE 10, no. 7: e0132177. 26136237 10.1371/journal.pone.0132177 PMC4489843

[brb370165-bib-0002] Altmann, P. , M. Ponleitner , P. S. Rommer , et al. 2021. “Seven Day Pre‐Analytical Stability of Serum and Plasma Neurofilament Light Chain.” Scientific Reports 11, no. 1: 11034. 34040118 10.1038/s41598-021-90639-z PMC8154890

[brb370165-bib-0003] Benkert, P. , S. Meier , S. Schaedelin , et al. 2022. “Serum Neurofilament Light Chain for Individual Prognostication of Disease Activity in People With Multiple Sclerosis: A Retrospective Modelling and Validation Study.” Lancet Neurology 21, no. 3: 246–257. 35182510 10.1016/S1474-4422(22)00009-6

[brb370165-bib-0004] Burtscher, M. , M. Flatz , and M. Faulhaber . 2004. “Prediction of Susceptibility to Acute Mountain Sickness by SaO_2_ Values During Short‐Term Exposure to Hypoxia.” High Altitude Medicine & Biology 5, no. 3: 335–340. 15453999 10.1089/ham.2004.5.335

[brb370165-bib-0005] Burtscher, M. , U. Hefti , and J. P. Hefti . 2021. “High‐Altitude Illnesses: Old Stories and New Insights Into the Pathophysiology, Treatment and Prevention.” Sports Medicine and Health Science 3, no. 2: 59–69. 35782163 10.1016/j.smhs.2021.04.001 PMC9219347

[brb370165-bib-0006] Burtscher, M. , M. Philadelphy , H. Gatterer , et al. 2019. “Physiological Responses in Humans Acutely Exposed to High Altitude (3480 m): Minute Ventilation and Oxygenation Are Predictive for the Development of Acute Mountain Sickness.” High Altitude Medicine & Biology 20, no. 2: 192–197. 30896981 10.1089/ham.2018.0143

[brb370165-bib-0007] Disanto, G. , C. Barro , P. Benkert , et al. 2017. “Serum Neurofilament Light: A Biomarker of Neuronal Damage in Multiple Sclerosis.” Annals of Neurology 81, no. 6: 857–870. 28512753 10.1002/ana.24954 PMC5519945

[brb370165-bib-0008] Fagenholz, P. J. , J. A. Gutman , A. F. Murray , V. E. Noble , C. A. Camargo Jr. , and N. S. Harris . 2009. “Optic Nerve Sheath Diameter Correlates With the Presence and Severity of Acute Mountain Sickness: Evidence for Increased Intracranial Pressure.” Journal of Applied Physiology 106, no. 4: 1207–1211. 19118159 10.1152/japplphysiol.01188.2007

[brb370165-bib-0009] Gattringer, T. , D. Pinter , C. Enzinger , et al. 2017. “Serum Neurofilament Light is Sensitive to Active Cerebral Small Vessel Disease.” Neurology 89, no. 20: 2108–2114. 29046363 10.1212/WNL.0000000000004645 PMC5711505

[brb370165-bib-0010] Hackett, P. H. , and R. C. Roach . 2001. “High‐Altitude Illness.” New England Journal of Medicine 345, no. 2: 107–114. 11450659 10.1056/NEJM200107123450206

[brb370165-bib-0011] Hackett, P. H. , P. R. Yarnell , R. Hill , K. Reynard , J. Heit , and J. McCormick . 1998. “High‐Altitude Cerebral Edema Evaluated With Magnetic Resonance Imaging: Clinical Correlation and Pathophysiology.” JAMA 280, no. 22: 1920–1925. 9851477 10.1001/jama.280.22.1920

[brb370165-bib-0012] Hoiland, R. L. , P. N. Ainslie , C. L. Wellington , et al. 2021. “Brain Hypoxia Is Associated With Neuroglial Injury in Humans Post‐Cardiac Arrest.” Circulation Research 129, no. 5: 583–597. 34287000 10.1161/CIRCRESAHA.121.319157 PMC8376277

[brb370165-bib-0013] Honigman, B. , M. K. Theis , J. Koziol‐McLain , et al. 1993. “Acute Mountain Sickness in a General Tourist Population at Moderate Altitudes.” Annals of Internal Medicine 118, no. 8: 587–592. 8452324 10.7326/0003-4819-118-8-199304150-00003

[brb370165-bib-0014] Jarius, S. , K. Ruprecht , B. Wildemann , et al. 2012. “Contrasting Disease Patterns in Seropositive and Seronegative Neuromyelitis Optica: A Multicentre Study of 175 Patients.” Journal of Neuroinflammation 9: 14. 22260418 10.1186/1742-2094-9-14 PMC3283476

[brb370165-bib-0015] Kallenberg, K. , D. M. Bailey , S. Christ , et al. 2007. “Magnetic Resonance Imaging Evidence of Cytotoxic Cerebral Edema in Acute Mountain Sickness.” Journal of Cerebral Blood Flow and Metabolism 27, no. 5: 1064–1071. 17024110 10.1038/sj.jcbfm.9600404

[brb370165-bib-0016] Kanaan, N. C. , G. S. Lipman , B. B. Constance , P. S. Holck , J. F. Preuss , and S. R. Williams . 2015. “Optic Nerve Sheath Diameter Increase on Ascent to High Altitude: Correlation With Acute Mountain Sickness.” Journal of Ultrasound in Medicine 34, no. 9: 1677–1682. 26269295 10.7863/ultra.15.14.10060

[brb370165-bib-0017] Khalil, M. , C. E. Teunissen , M. Otto , et al. 2018. “Neurofilaments as Biomarkers in Neurological Disorders.” Nature Reviews Neurology 14, no. 10: 577–589. 30171200 10.1038/s41582-018-0058-z

[brb370165-bib-0018] Luks, A. M. , E. R. Swenson , and P. Bärtsch . 2017. “Acute High‐Altitude Sickness.” European Respiratory Review 26, no. 143: 160096. 10.1183/16000617.0096-2016. 28143879 PMC9488514

[brb370165-bib-0019] Mairer, K. , M. Wille , T. Bucher , and M. Burtscher . 2009. “Prevalence of Acute Mountain Sickness in the Eastern Alps.” High Altitude Medicine & Biology 10, no. 3: 239–245. 19775213 10.1089/ham.2008.1091

[brb370165-bib-0020] Netzer, N. , K. Strohl , M. Faulhaber , H. Gatterer , and M. Burtscher . 2013. “Hypoxia‐Related Altitude Illnesses.” Journal of Travel Medicine 20, no. 4: 247–255. 23809076 10.1111/jtm.12017

[brb370165-bib-0021] Novakova, L. , H. Zetterberg , P. Sundström , et al. 2017. “Monitoring Disease Activity in Multiple Sclerosis Using Serum Neurofilament Light Protein.” Neurology 89, no. 22: 2230–2237. 29079686 10.1212/WNL.0000000000004683 PMC5705244

[brb370165-bib-0022] Pedersen, A. , T. M. Stanne , S. Nilsson , et al. 2019. “Circulating Neurofilament Light in Ischemic Stroke: Temporal Profile and Outcome Prediction.” Journal of Neurology 266, no. 11: 2796–2806. 31375988 10.1007/s00415-019-09477-9 PMC6803587

[brb370165-bib-0023] Rissin, D. M. , C. W. Kan , T. G. Campbell , et al. 2010. “Single‐Molecule Enzyme‐Linked Immunosorbent Assay Detects Serum Proteins at Subfemtomolar Concentrations.” Nature Biotechnology 28, no. 6: 595–599. 10.1038/nbt.1641 PMC2919230 20495550

[brb370165-bib-0024] Roach, R. C. , P. H. Hackett , O. Oelz , et al. 2018. “The 2018 Lake Louise Acute Mountain Sickness Score.” High Altitude Medicine & Biology 19, no. 1: 4–6. 29583031 10.1089/ham.2017.0164 PMC6191821

[brb370165-bib-0025] Ross, R. T. 1985. “The Random Nature of Cerebral Mountain Sickness.” Lancet 1, no. 8435: 990–991. 2859454 10.1016/s0140-6736(85)91771-4

[brb370165-bib-0026] Sareban, M. , M. M. Berger , D. Pinter , et al. 2021. “Serum Neurofilament Level Increases After Ascent to 4559 m but Is Not Related to Acute Mountain Sickness.” European Journal of Neurology 28, no. 3: 1004–1008. 33095952 10.1111/ene.14606 PMC7898504

[brb370165-bib-0027] Schneider, M. , D. Bernasch , J. Weymann , R. Holle , and P. Bartsch . 2002. “Acute Mountain Sickness: Influence of Susceptibility, Preexposure, and Ascent Rate.” Medicine and Science in Sports and Exercise 34, no. 12: 1886–1891. 12471292 10.1097/00005768-200212000-00005

[brb370165-bib-0028] Schommer, K. , K. Kallenberg , K. Lutz , P. Bärtsch , and M. Knauth . 2013. “Hemosiderin Deposition in the Brain as Footprint of High‐Altitude Cerebral Edema.” Neurology 81, no. 20: 1776–1779. 24107867 10.1212/01.wnl.0000435563.84986.78

[brb370165-bib-0029] Shahim, P. , M. Gren , V. Liman , et al. 2016. “Serum Neurofilament Light Protein Predicts Clinical Outcome in Traumatic Brain Injury.” Scientific Reports 6: 36791. 27819296 10.1038/srep36791 PMC5098187

[brb370165-bib-0030] Shahim, P. , H. Zetterberg , Y. Tegner , and K. Blennow . 2017. “Serum Neurofilament Light as a Biomarker for Mild Traumatic Brain Injury in Contact Sports.” Neurology 88, no. 19: 1788–1794. 28404801 10.1212/WNL.0000000000003912 PMC5419986

[brb370165-bib-0031] Tiedt, S. , M. Duering , C. Barro , et al. 2018. “Serum Neurofilament Light: A Biomarker of Neuroaxonal Injury After Ischemic Stroke.” Neurology 91, no. 14: e1338–e1347. 30217937 10.1212/WNL.0000000000006282

[brb370165-bib-0032] Treml, B. , A. Kleinsasser , T. Hell , H. Knotzer , M. Wille , and M. Burtscher . 2020. “Carry‐Over Quality of Pre‐Acclimatization to Altitude Elicited by Intermittent Hypoxia: A Participant‐Blinded, Randomized Controlled Trial on Antedated Acclimatization to Altitude.” Frontiers in Physiology 11: 531. 32547414 10.3389/fphys.2020.00531 PMC7272681

[brb370165-bib-0033] Wilson, M. H. , S. Newman , and C. H. Imray . 2009. “The Cerebral Effects of Ascent to High Altitudes.” Lancet Neurology 8, no. 2: 175–191. 19161909 10.1016/S1474-4422(09)70014-6

