# Supplementary material for: Neurofilament Light Chain Is Associated With Acute Mountain Sickness
Source: Brain Behav. 2024 Nov 17;14(11):e70165. doi: 10.1002/brb3.70165 (PMC11570677; doi:10.1002/brb3.70165)
Supplement: Supplementary file 1 — Supporting Information [file BRB3-14-e70165-s001.docx]

**Supplemental Table 1**. Differences of pNfL levels according to pre-acclimatization group.

|  | **Control Group** | **Acclimatization Group** | **p-value** |
| --- | --- | --- | --- |
| pNfL at M3 (pg/ml) | 5.20 [4.34-7.84] | 6.89 [5.03-8.77] | 0.203 |
| pNfL at M4 (pg/ml) | 6.53 [4.18-7.33] | 6.89 [5.21-8.83] | 0.242 |
| pNfL increase C2^a^ | 0.16 [-1.34-1.87] | 0.18 [-0.90-1.40] | 0.884 |

Legend:

All values are depicted as median [IQR]. Group comparisons were performed by Mann-Whitney U test. p-values<0.05 are considered statistically significant and are marked bold.

^a^ indicates increase after exposure to simulated high altitude.

M3-4=Measurement3-4, pNfL=plasma Neurofilament Light.
